# Supplementary material for: backbone: An R package to extract network backbones
Source: PLoS One. 2022 May 31;17(5):e0269137. doi: 10.1371/journal.pone.0269137 (PMC9154188; doi:10.1371/journal.pone.0269137)
Supplement: Attachment — Submitted filename: Memo.pdf [file pone.0269137.s001.pdf]

Summary of revisions to:
"backbone: An R package to extract network backbones" (PONE-D-22-07882)

Dear Drs. Cherifi, Rosato, Ghalmane, and anonymous reviewer,

Thank you for these very helpful comments, and for the opportunity to revise and resubmit. Below we provide detailed responses to each of your comments. In the revised manuscript, key revisions are highlighted using red text. However, because `verbatim` LaTeX text cannot be colored red, revisions to the displayed code are not highlighted.

1 PLOS-related revisions

- The original submission described `backbone v2.0.0`, while the revised manuscript describes `backbone v2.1.0`. This new version is currently available as a 'beta' release via GitHub and can be installed in R using `devtools::install_github("zpneal/backbone", ref = "devel")`. Pending acceptance of this manuscript, it will become the 'alpha' release and will be publicly distributed via CRAN.

- Following PLOS guidelines, I have removed the funding statement from the acknowledgements section. The existing funding statement ("ZN received funding from the National Science Foundation (www.nsf.gov), awards #1851625 and #2016320. The funders had no role in study design, data collection and analysis, decision to publish, or preparation of the manuscript.") is correct.

- To facilitate peer review, I have submitted a version that is formatted similarly to the original (e.g., figures are displayed in the main text). However, I have prepared a version of the manuscript that follows the PLOS figure guidelines (e.g., no figures displayed), which I can provide if this manuscript is accepted.

2 Reviewer #1 (anonymous)

2.1 "I love many things about this paper/package, among them being a built-in way to do multiple test corrections, or the possibility of returning signed backbones."

I am very happy to hear that you enjoyed the paper and package, including the new MTC and signed backbone capabilities.

2.2 "The limitation section is honest about the lack of implementation for many methods used in the literature. I feel the author really should add the following to the list: Slater (2009); Grady et al. (2012); Gemmetto et al. (2017); Coscia and Neffke (2017); Šubelj (2018). If these methods are cited, it reassures the reader that they are in the radar and will be added soon. Many of the papers I mention have dozens of citations and they are widely used, a few works even for unweighted networks."

Thank you for bringing these works to my attention. I have added most of these citations to the limitations section, and will add them to my list of methods to be implemented in future versions. I have omitted a reference to Grady et al. (2012), whose only mention of backbone extraction is

to Serrano et al. (2009), which is already cited.

2.3 "As of now, I can say that this paper is good, and the backbone package is good, but I'm not using it, because most of the times I use backboning methods that are not implemented in it. If they are not part of the planned development, I don't think I will make the investment of learning to use the package."

This version of the backbone package introduced a basic framework for structural and statistical backbone models for projections, weighted networks, and unweighted networks. Now that the framework exists, my plan is to incrementally implement additional models. I appreciate the recommendations above, and hope that soon the package will have sufficient functionality that you'll find it useful in your own work.

2.4 "In the introduction the author says that the backbone is an unweighted network, but the paper also mentions that it can consider "p-values as edge weights". However, some methods can re-normalize weights (like, e.g., Slate or Grady) or use z- or t-scores. I'd use a different language in the introduction, because as of now it seems the package returns exclusively unweighted backbones, which appears to be a severe limitation."

The introduction is correct that the backbones generated by the `backbone` package are unweighted networks. The package (and models it implements) are designed to solve the challenges of analyzing dense and weighted networks by extracting a sparse and unweighted backbone.

There are indeed many methods for modifying, transforming, or normalizing edge weights in a weighted network. Although these methods can be quite useful, considering them goes beyond the scope of this package and paper because they are designed to solve a different problem. They do not solve the challenges associated with analyzing weighted networks because they yield networks that are still weighted.

I agree that it was confusing to suggest that p-values could be treated like edge weights. Such an approach is also inconsistent with statistical practice (Wasserstein and Lazar, 2016). Therefore, I have removed this claim and the entire subsection on 'Interpreting p-values.' This section has been replaced with a section titled 'Correcting p-values' that narrowly deals with issues of multiple independent hypothesis tests.

2.5 "In the background section the author fails to mention that often, especially in graph theory, edge weights might represent costs, and not capacities. If a network has such weights, a backbone method that assumes edge weights being capacities would select the wrong edges for the backbone. This problem should be mentioned to make sure the user understands the assumption baked up in the package – and perhaps having a function "costs2capacities" in the package could be useful."

Thank you for reminding me of this important point. I have clarified the scope of the discussion at the beginning of the 'background' subsection in the section on 'backbones of weighted networks.'

I agree that providing a function that would allow users to transform costs into capacities, and

vice versa, would be a useful addition and expand the applicability of the package. In practice, this could be challenging because costs and capacities do not always have a simple or known relationship, however I will certainly explore opportunities for extending `backbone` in this direction.

2.6 "An important information the package should also return is the fraction of nodes dropped from the backbone because they became isolates – they have no edges in the backbone. Global threshold and disparity filter tend to isolate lots of nodes, while some of the cited methods don't."

This is a great suggestion. I have modified the narrative text returned when the `narrative = TRUE` option is used. The text now includes a sentence of the form: "This reduced the number of edges by XX%, and reduced the number of connected nodes by YY%." For example, the narrative text describing the application of the disparity filter to the example airline data now reports "This reduced the number of edges by 91.4%, and reduced the number of connected nodes by 14.7%." The manuscript has been updated to show the new narrative text outputs from each of the examples.

3 Reviewer #2 (Antonio Rosato)

3.1 "The presentation is very clear with few relevant examples taken from public sources and made available also through the author's github repository. The meaning and importance of selected relevant parameters are addressed in good detail. Overall I think this will be a very useful publication especially in the context of training students/postdocs in the analysis of network as well as to enable less expert statisticians to make some fundamental analysis."

I'm happy to hear that you found the presentation clear, and that you appreciated the examples. One goal in this paper was to present the material in a sufficiently accessible style that it would be useful for students and newcomers to backbone methods.

3.2 "the abstract mentions that 'This paper introduces [...] the 'backbone' package for R,'. I am not sure what is meant here - the package has already been published elsewhere.

To clarify, I have revised the abstract as: "This paper introduces and demonstrates a substantially expanded version of the `backbone` package for R, which now provides methods..."

3.3 "in light of their relevance throughout the paper, the author might want to explain in one paragraph what are logarithmic degree distribution plots and the associated parameters"

The degree distribution plots were not intended to be a significant feature of the analysis, and some have suggested that investigating degree distributions graphically using logarithmic plots can sometimes be misleading. Therefore, in this revision I have eliminated the plots. I have retained the estimated degree scaling parameters, but have added additional text explaining their interpretation.

3.4 "to support adoption by teachers, it would be quite useful if the author provided other example datasets that are not demonstrated in the paper but could be analysed fruitfully using the 'backbone' package (e.g. in a hands-on lab session)"

The current paper provides three empirical datasets drawn from three different contexts (transportation, politics, friendship). While providing even more datasets could be useful for instructional purposes, doing so would go beyond the scope of a single manuscript, and might be more appropriate as supplementary material in the development of a textbook. However, throughout the manuscript and especially in the 'applications' section I point interested readers to papers that use these types of models and provide their datasets.

4 Reviewer #3 (Zakariya Ghalmane)

4.1 "This article is certainly relevant for the readership of PLOS one and I am confident that, once the authors will have addressed the suggestions below, it will be a suitable article for publication."

I am happy to hear that you found the work relevant to PLOS readers.

4.2 The authors seem to be unaware of recent works on backbone extraction methods using information about the community structure of the network worth quoting: Ghalmane et al. (2021, 2020)"

Thank you for bringing these works to my attention. In the revision, I have incorporated a citation to Ghalmane et al. (2021). I have chosen not to cite Ghalmane et al. (2020) because Ghalmane et al. (2021) (newly cited) and Rajeh et al. (2022) (cited already) introduce methods that they find outperform the 'overlapping nodes ego backbone' and 'overlapping nodes and hubs backbone'.

References

Michele Coscia and Frank MH Neffke. Network backboning with noisy data. In *2017 IEEE 33rd International Conference on Data Engineering (ICDE)*, pages 425–436. IEEE, 2017. doi: 10.1109/ICDE.2017.100.

Valerio Gemmetto, Alessio Cardillo, and Diego Garlaschelli. Irreducible network backbones: unbiased graph filtering via maximum entropy. *arXiv preprint arXiv:1706.00230*, 2017. doi: 10.48550/arXiv.1706.00230.

Zakariya Ghalmane, Chantal Cherifi, Hocine Cherifi, and Mohammed El Hassouni. Extracting backbones in weighted modular complex networks. *Scientific Reports*, 10(1):1–18, 2020. doi: 10.1038/s41598-020-71876-0.

Zakariya Ghalmane, Chantal Cherifi, Hocine Cherifi, and Mohammed El Hassouni. Extracting modular-based backbones in weighted networks. *Information Sciences*, 576:454–474, 2021. doi: 10.1016/j.ins.2021.06.087.

Daniel Grady, Christian Thiemann, and Dirk Brockmann. Robust classification of salient links in complex networks. *Nature communications*, 3(1):1–10, 2012. doi: 10.1038/ncomms1847.

Stephany Rajeh, Marinette Savonnet, Eric Leclercq, and Hocine Cherifi. Modularity-based backbone extraction in weighted complex networks. In Pedro Ribeiro, Fernando Silva, José Fernando Mendes, and Rosário Laureano, editors, *Network Science*, pages 67–79, Cham, 2022. Springer International Publishing. doi: 10.1007/978-3-030-97240-0_6.

M Ángeles Serrano, Marián Boguná, and Alessandro Vespignani. Extracting the multiscale backbone of complex weighted networks. *Proceedings of the National Academy of Sciences*, 106 (16):6483–6488, 2009. doi: 10.1073/pnas.0808904106.

Paul B Slater. A two-stage algorithm for extracting the multiscale backbone of complex weighted networks. *Proceedings of the National Academy of Sciences*, 106(26):E66–E66, 2009. doi: 10.1073/pnas.0904725106.

Lovro Šubelj. Convex skeletons of complex networks. *Journal of The Royal Society Interface*, 15 (145):20180422, 2018. doi: 10.1098/rsif.2018.0422.

Ronald L Wasserstein and Nicole A Lazar. The asa statement on p-values: context, process, and purpose, 2016.
